# Supplementary material for: An open-source framework for large-scale, flexible evaluation of biomedical text mining systems
Source: J Biomed Discov Collab. 2008 Jan 29;3:1. doi: 10.1186/1747-5333-3-1 (PMC2276192; doi:10.1186/1747-5333-3-1)
Supplement: Additional file 1 — Evaluation platform source code. The additional file contains the software evaluation framework discussed in this paper. The most current release can be downloaded from SourceForge.net [1]. See the accompanying README file for instructions on installation and use. Also included in the distribution is a collection of UIMA wrappers for some commonly used BioNLP tools and annotated corpora. [file 1747-5333-3-1-S1.zip › bionlp_uima_repository-1.0.1/docs/api/edu/uchsc/ccp/uima/util/UIMA_Annotation_Util.html]

UIMA\_Annotation\_Util (Center for Computational Pharmacology Util/UIMA API)


|  |  |  |
| --- | --- | --- |
| **Overview** \| **Package** \| **Class** \| **Tree** \| **Deprecated** \| **Index** \| **Help** | |  |
| PREV CLASS   **NEXT CLASS** | **FRAMES**    **NO FRAMES**     **All Classes** |  |
| SUMMARY: NESTED \| FIELD \| CONSTR \| METHOD | DETAIL: FIELD \| CONSTR \| METHOD |  |


---


## edu.uchsc.ccp.uima.util Class UIMA\_Annotation\_Util

```
java.lang.Object
  edu.uchsc.ccp.uima.util.UIMA_Annotation_Util
```

---

``` public class UIMA_Annotation_Util extends Object ```

---

| **Field Summary** | |
| --- | --- |
| `static boolean` | `DEBUG` |


**Constructor Summary**

`UIMA_Annotation_Util()`


| **Method Summary** | |
| --- | --- |
| `static boolean` | `overlaps(CCPTextAnnotation ccpTA1, CCPTextAnnotation ccpTA2)` |
| `static void` | `removeLeadingAndTrailingBlankLines(org.apache.uima.jcas.tcas.Annotation annotation)` |
| `static void` | `trimCCPTextAnnotation(CCPTextAnnotation ccpTA)`             trims spaces and   from ends of a CCPTextAnnotation |

**Methods inherited from class java.lang.Object**

`clone, equals, finalize, getClass, hashCode, notify, notifyAll, toString, wait, wait, wait`

**Field Detail**

### DEBUG

```
public static final boolean DEBUG
```

**See Also:**: Constant Field Values


**Constructor Detail**

### UIMA\_Annotation\_Util

```
public UIMA_Annotation_Util()
```


**Method Detail**

### removeLeadingAndTrailingBlankLines

```
public static void removeLeadingAndTrailingBlankLines(org.apache.uima.jcas.tcas.Annotation annotation)
```

---


### trimCCPTextAnnotation

```
public static void trimCCPTextAnnotation(CCPTextAnnotation ccpTA)
                                  throws org.apache.uima.cas.CASException
```

:   trims spaces and   from ends of a CCPTextAnnotation

    :   **Parameters:**: `ccpTA` - **Throws:**: `org.apache.uima.cas.CASException`

---


### overlaps

```
public static boolean overlaps(CCPTextAnnotation ccpTA1,
                               CCPTextAnnotation ccpTA2)
```


---


|  |  |  |
| --- | --- | --- |
| **Overview** \| **Package** \| **Class** \| **Tree** \| **Deprecated** \| **Index** \| **Help** | |  |
| PREV CLASS   **NEXT CLASS** | **FRAMES**    **NO FRAMES**     **All Classes** |  |
| SUMMARY: NESTED \| FIELD \| CONSTR \| METHOD | DETAIL: FIELD \| CONSTR \| METHOD |  |


---
